# Supplementary material for: Coordinated macrophage and T cell interactions mediate response to checkpoint blockade in colorectal cancer
Source: bioRxiv. 2025 Feb 17:2025.02.12.637954. Preprint. [Version 1] doi: 10.1101/2025.02.12.637954 (PMC11870396; doi:10.1101/2025.02.12.637954)
Supplement: Supplement 1 [file NIHPP2025.02.12.637954v1-supplement-1.pdf]

bioRxiv preprint doi: https://doi.org/10.1101/2025.02.12.637954; this version posted February 17, 2025. The copyright holder for this preprint (which was not certified by peer review) is the author/funder, who has granted bioRxiv a license to display the preprint in perpetuity. It is made available under aCC-BY 4.0 International license.

Supplementary data

Treatment / tumor elimination rate (%)	CT26 MSH2KO (%)	CT26 WT (%)	4T1 MSH2KO (%)	B16F10 MSH2KO (%)
**no antibody**	0	0	0	0
**isotypes**	0	0	0	0
**anti PD1**	14	0	0	0
**anti TIM3**	0	0	0	0
**anti TIGIT**	0	0	0	0

bioRxiv preprint doi: https://doi.org/10.1101/2025.02.12.637954; this version posted February 17, 2025. The copyright holder for this preprint (which was not certified by peer review) is the author/funder, who has granted bioRxiv a license to display the preprint in perpetuity. It is made available under aCC-BY 4.0 International license.

**anti LAG3**	0	0	0	0
**anti CTLA4**	0	0	0	0
**anti TREM2**	0	0	0	0
**anti IFITM**	10	0	0	0
**anti PD1 TIM3**	25	0	0	0
**anti PD1 TIGIT**	12	10	0	0
**anti PD1 LAG3**	59	20	0	0
**anti PD1 CTLA4 LAG3**	71	50	17	17
**anti PD1 CTLA4 LAG3 TREM2**	100	73	33.33	15

Table 1) Percentage of complete tumor elimination in mice according to treatment
